# Suppression of R5-type of HIV-1 in CD4^+^ NKT cells by Vδ1^+^ T cells activated by flavonoid glycosides, hesperidin and linarin

**DOI:** 10.1038/s41598-019-40587-6

**Published:** 2019-05-17

**Authors:** Michiyuki Yonekawa, Masumi Shimizu, Atsushi Kaneko, Jiro Matsumura, Hidemi Takahashi

**Affiliations:** Department of Microbiology and Immunology, Nippon Medical School, Tokyo, Japan

**Keywords:** Innate lymphoid cells, HIV infections

## Abstract

We established transfectants expressing T cell receptors (TCRs) either for Vγ1 and Vδ1 (1C116) or for Vγ2 and Vδ2 (2C21) using the TCR-deficient Jurkat T cell line J.RT3-T3.5. The amount of IL-2 secreted from these γδ T cell clones accurately indicated TCR-dependent stimulation. Clone 2C21 was specifically stimulated by previously reported ligands for Vγ2Vδ2 (Vδ2)-TCR such as isopentenyl pyrophospate (IPP), ethylamine, or risedronate. In contrast, clone 1C116 was strongly stimulated through the Vγ1Vδ1 (Vδ1)-TCR by flavonoid glycosides such as hesperidin and linarin, having both rutinose at the A ring and methoxy (-OCH3) substitution at the B ring. Additionally, hesperidin and linarin showed stimulatory activity for peripheral blood mononuclear cell (PBMC)-derived T cells expressing Vδ1-TCR; these activated Vδ1^+^ T cells also secreted IL-5, IL-13, MIP-1α, MIP-1β and RANTES. Such PBMC-derived Vδ1^+^ T cells stimulated by hesperidin and linarin suppressed R5-HIV-1-NL(AD8) viral replication in CD4^+^ NKT cells in a dose-dependent manner. To the best of our knowledge, this is the first demonstration that flavonoid glycosides activate functional Vδ1^+^ T cells.

## Introduction

The development of anti-HIV-1 drugs, particularly the newer class of anti-retroviral drugs such as integrase inhibitors (IN), raltegravir, elvitegravir, and dolutegravir, now permits HIV-1 to be successfully eradicated from the circulating blood of HIV-1-infected individuals^1^. Nevertheless, in most cases, HIV-1 virions still re-emerge within a few months after interruption of anti-retroviral therapy (ART)^2,3^. These findings suggest that immunity to HIV-1 able to prevent the generation of circulating virus does not arise in most ART-treated infected individuals. Although free virions and HIV-1-p24 antigen (p24)-positive cells were not seen in the blood of patients receiving current effective ART, proviral DNA and HIV-1 p24 antigen could still be detected in the ileum of the same patient. Indeed, we have recently confirmed that Vα24^+^ natural killer T (NKT) cells^4^ were the major p24-positive cells among the HIV-1-infected CD4^+^ cells in the ileum samples, suggesting that the innate NKT cells in the mucosal compartment are the most likely candidates for the origin of the HIV-1 that emerges after ART is interrupted^5^.

The majority of emergent HIV-1 after interrupting ART is macrophage-tropic and infects CD4^+^, CCR5-expressing cells (R5-tropic) rather than CXCR4 expressing CD4^+^ T cells (X4-tropic)^5^. Additionally, we have verified that CD4^+^ NKT cells from peripheral blood mononuclear cells (PBMCs) predominantly express CCR5 rather than CXCR4 and are infected with R5-tropic HIV-1, such as the NL(AD8) isolate, which expands efficiently in the CD4^+^ NKT cells^5,6^, as has been reported^7^. In addition, we found that CD8αα^+^ but not CD8αβ^+^ T cells have the ability to inhibit R5-tropic HIV-1 replication in the CD4^+^ NKT cells and confirmed that replication of the NL(AD8) isolate in the CD4^+^ NKT cells was efficiently suppressed by CD8αα^+^ γδ T cells, in particular Vγ1Vδ1(Vδ1)-T cell receptor (TCR)-expressing γδ T cells, mainly through chemokines such as macrophage inflammatory proteins 1alpha (MIP-1α) and MIP-1β and RANTES^6^. These results suggest that CD8αα^+^ Vδ1^+^ T cells may contribute to control of R5-tropic HIV-1 replication in persistently infected CD4^+^ NKT cells.

However, the Vδ1^+^ T cells were obtained from freshly-isolated PBMC-derived T cells, which were variably activated by the procedure of their purification process with specific antibodies. Thus, the suppressing effect of the Vδ1^+^ T cells on HIV-1 replication in CD4^+^ NKT cells was uneven. Therefore, to examine more stable effects of Vδ1^+^ T cells, we used here resting Vδ1^+^ T cells purified according to a procedure by Shamshiev *et al*.^8^ as responders to confirm the suppressing effects.

Human γδ T cells consist mainly of two distinct subsets, Vδ1-TCR-expressing Vδ1^+^ T cells and Vδ2-TCR-expressing Vδ2^+^ T cells. These lymphocytes play important roles in bridging innate and adaptive immunity, but their recognition mechanisms remain poorly understood. Approximately 70% of T lymphocytes express the Vδ2-TCR and can be expanded and activated by phospho-antigens such as the cholesterol biosynthesis-related substance, isopentenyl pyrophosphate (IPP)^9^, or synthetic bisphosphonates, such as pamidronate disodium and zoledronic acid^10^, as well as alkylamines such as ethylamine^11^. In contrast, the Vδ1^+^ T cell subset is predominantly present in the intestinal epithelia and responds to MICA and MICB (MHC class I chain-related, A and B; MIC) self-antigens, as well as CD1-molecule related lipid antigens^12,13^, mediating responses to tumorigenesis or viral infection^14^. In contrast to αβ TCRs, which require antigen processing and subsequent presentation antigenic peptides by MHC molecules, γδ TCRs are believed to recognize antigens directly^15–17^.

In the present study, to identify antigenic ligands for the Vδ1-TCR, we first established two distinct clones from human γδT cell lines with stable proliferating ability and that express either Vγ1 and Vδ1 or Vγ2 and Vδ2 TCR. Full-length cDNAs encoding the TCR-γ1/TCR-δ1 chain or the TCR-γ2/TCR-δ2 chain were obtained from each T cell clone and transfected as pairs into a TCR-deficient Jurkat T cell line, J.RT3-T3.5^18^. We successfully established two distinct transfected clones expressing either Vγ1 and Vδ1 (1C116) or Vγ2 and Vδ2 (2C21). After confirming that clone 2C21 specifically responded to produce IL-2 upon stimulation by IPP, alkylamines such as ethylamine, and risedronate, we exposed the other clone 1C116 to various candidate antigenic molecules, including phytochemicals, such as alkaloids (compounds containing nitrogen), terpenoids (compounds derived from C5 isoprene units) and phenolics (compounds having aromaticity).

Among the flavonoids, we discovered two compounds, hesperidin and linarin, with very similar structures (C6-C3-C6 structure) containing a flavonoid glycoside that will specifically stimulate clone 1C116. Such flavonoid glycosides are well-known for their antioxidant, anti-inflammatory, anti-thrombogenic, anti-arteriosclerosis and anti-carcinogenic properties. Here, we demonstrate that both flavonoid glycosides suppressed the replication of R5-type HIV-1 in CD4^+^ NKT cells through the activation of Vδ1-TCR-bearing resting Vδ1^+^ T cells.

## Results

### Establishment of two distinct transfectants expressing T cell receptors (TCRs) either for Vγ1 and Vδ1 (1C116) or for Vγ2 and Vδ2 (2C21) from TCR- deficient Jurkat T cell line J.RT3-T3.5

As described in the Methods section, we established single cell clones bearing either Vδ1-TCR or Vδ2-TCR with stable proliferation capacity. Full-length cDNAs encoding both TCR-γ1 and TCR-δ1 chains were isolated from a Vδ1^+^ T cell clone. Similarly, the full-length cDNAs encoding both TCR-γ2 and TCR-δ2 chains were obtained from a Vδ2^+^ T cell clone (Supplementary Fig. S1). These plasmids were then doubly transfected into a TCR-deficient Jurkat T cell line, J.RT3-T3.5, generating two distinct transfected clones expressing either Vγ1 and Vδ1 (1C116) or Vγ2 and Vδ2 (2C21). These J.RT3-T3.5-derived transfectants were maintained in the presence of 1 mg/mL G418 sulfate and 0.5 mg/mL hygromycin B to maintain selection of γδ TCR-bearing cells.

**Figure 1:**
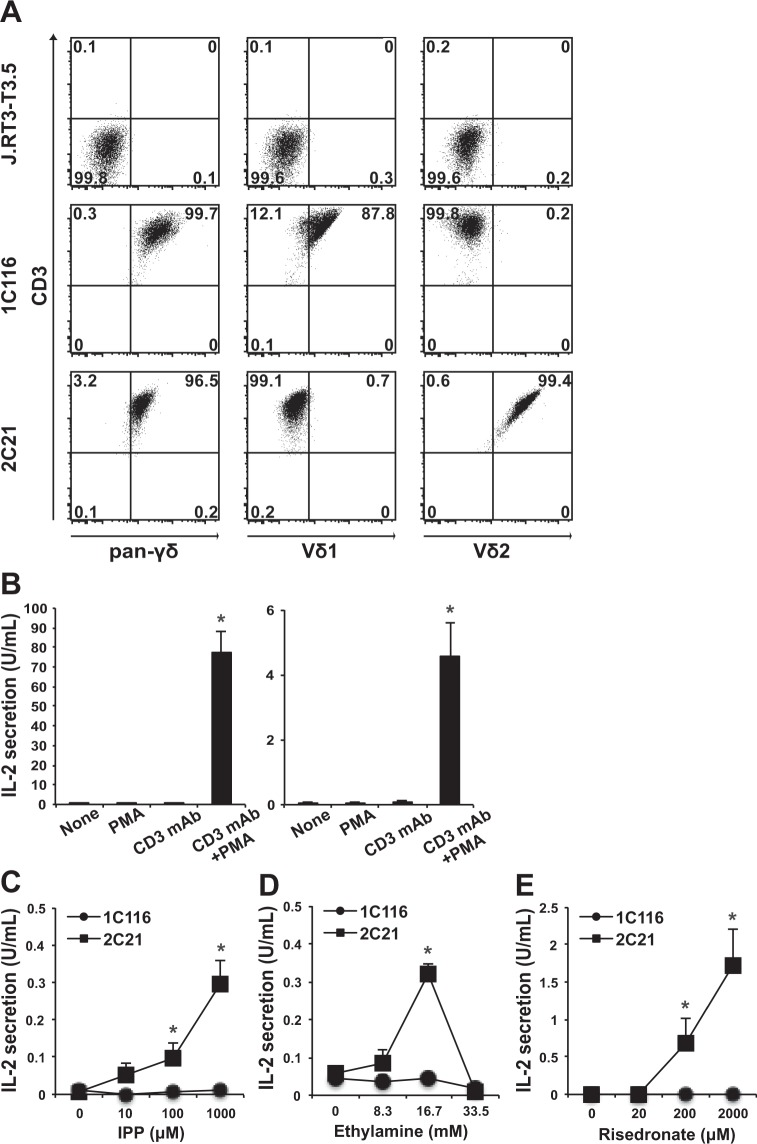
Flow cytometric analysis and IL-2 secretion of clones 1C116 and 2C21. (**A**) J.RT3-T3.5 (upper), Vδ1-TCR transfected clone 1C116 (middle) and Vδ2-TCR transfected clone 2C21 (lower) were stained with the antibodies. The experiment was repeated three times, and representative data are shown. (**B**) Clones 1C116 (left) and 2C21 (right) were stimulated with PMA, plate-bounded anti-CD3 antibody (CD3 mAb), or PMA and plate-bounded CD3 mAb for 24 h. The clones were stimulated with (**C**) IPP, (**D**) ethylamine or (**E**) risedronate for 24 h, and the amount of IL-2 in the culture supernatants was measured. The data are expressed as the mean + SEM of three independent experiments. *P < 0.05 ANOVA with Dunnett’s test.

As shown in Fig. 1A, clone 1 C116 was stained with monoclonal antibodies (mAbs) for pan-γδ and Vδ1 but not Vδ2, whereas clone 2C21 was stained with pan-γδ and Vδ2 but not Vδ1. Since both clones express CD3, when they are stimulated through the TCR, they can secrete IL-2 in the presence of PMA (Fig. 1B). Therefore, the amount of IL-2 secreted from these γδ T cell clones seems to be an excellent indicator for TCR-dependent stimulation.

Isopentenyl pyrophospate (IPP) is known to stimulate functional responses from Vδ2^+^ T cells^9,19^, although Vavassori *et al*. has been reported that Vδ2^+^ cells are not directly stimulated by IPP^20^. However, Bukowski, *et al*. reported^19^ that an IPP-like phosphoantigen MEP directly stimulated Vγ2/Vδ2^+^ TCRs-tranfected Jurkat T cells such as clone 2C21. On the basis of these findings, we examined whether IPP stimulates clone 2C21 to secrete IL-2. As shown in Fig. 1C, we observed that clone 2C21 secreted IL-2 when stimulated with IPP, whereas clone 1C116 did not. Vδ2^+^ T cells can also be activated by other molecules, like various alkylamines such as ethylamine, propylamine or butylamine^11^, or amino-bisphosphonates used for the treatment of osteoporosis^21^. Similarly, clone 2C21 was stimulated to secrete IL-2 by ethylamine in a dose-dependent manner until 16.7 mM, but was not stimulated by methylamine while more than 33.5 mM of ethylamine were toxic and the secretion of IL-2 by clone 2C21 was abrogated (Fig. 1D). Moreover, clone 2C21 was functionally activated by amino-bisphosphonate such as risedronate (Fig. 1E). These findings indicate that clone 2C21 but not clone 1C116 is specifically stimulated by these various molecules acting through the Vδ2-TCR.

### Screening of various phytochemicals for their stimulatory activity on clones 1C116 or 2C21 to secrete IL-2

It is well known that some plant metabolites have biological activities. Recently, such natural compounds have been called “phytochemicals”^22^. According to their metabolic pathways and their chemical structures, phytochemicals are mainly classified into alkaloids (compounds containing nitrogen), terpenoids (compounds derived from C5 isoprene units) and phenolics (compounds having aromaticity). Among phytochemicals, the phenolics make up a large group and include one of the most ubiquitous botanical products called flavonoids (compounds having C6-C3-C6 structure), which are well-known for their many beneficial properties, showing antioxidant, anti-inflammatory, anti-thrombogenic, anti-arteriosclerosis and anti-carcinogenic activity, and they are considered to be important resources for drug discovery^23^.

**Figure 2:**
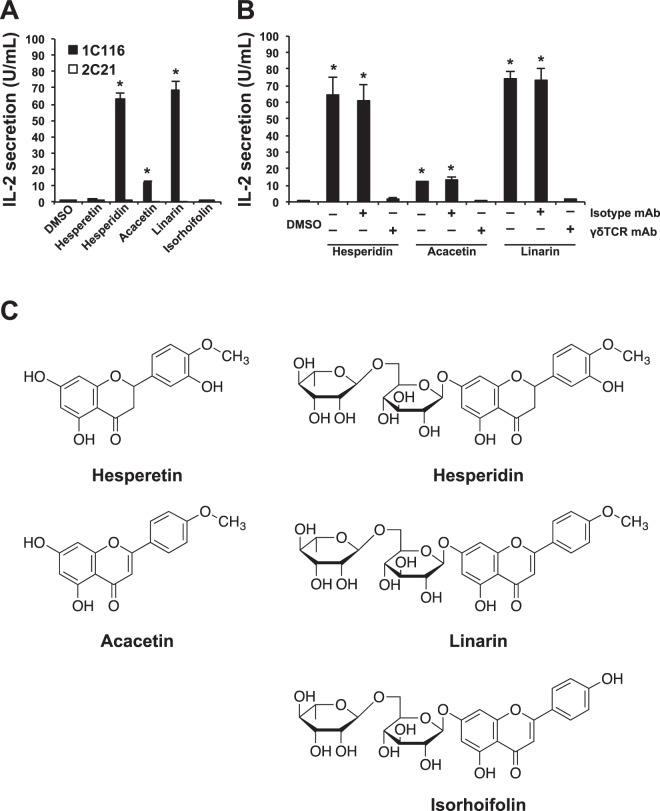
Effects of flavonoid glycosides on clone 1C116 through Vδ1-TCR. (**A**) Clones 1C116 (closed square) and 2C21 (open square) were stimulated with flavonoid glycosides, such as hesperidin and linarin, and with their analogs, such as hesperetin, acacetin and isorhoifolin, for 24 h, and the amount of IL-2 in the culture supernatants was measured. (**B**) Clone 1C116 (closed square) was stimulated with hesperidin, acacetin or linarin for 24 h either in the absence of blocking antibody or in the presence of anti-pan-γδ (γδTCR mAb) or their isotype control (Isotype mAb). The data are expressed as the mean + SEM of three independent experiments. *P < 0.05 ANOVA with Dunnett’s test performed in triplicate. (**C**) Chemical structures of flavonoid glycosides and their analogs that were used in this experiment. Among these compounds, only hesperidin and linarin have the rutinose (6-O-α-L-rhamnosyl-D-glucose) substitution at the A ring together with the methoxy (-OCH3) substitution at the B ring.

To examine whether flavonoids might act in part through immune cells, we screened several compounds for their capacity to activate clones 1C116 and 2C21. As shown in Fig. 2A and Table 1, some flavonoids stimulated clone 1C116 but not clone 2C21 to secrete large amounts of IL-2. In particular, flavonoids having a rutinose disaccharide such as 6-O-α-L-rhamnosyl-D-glucose on the A ring together with methoxy (-OCH3) substitution on the B ring, such as hesperidin and linarin, strongly induced clone 1C116 to secrete IL-2. The fact that such stimulatory potency was totally abrogated by the treatment with anti-γδ TCR-specific antibody strongly indicates that the recognition of the flavonoid glycoside was initiated through the Vδ1-TCR (Fig. 2B). Moreover, when treated with rutinose-deficient hesperidin (hesperetin) or rutinose-deficient linarin (acacetin), IL-2 secretion from clone 1C116 was not detected or weakly detected (about one-sixth of linarin), respectively. Additionally, when treated with flavonoids that have a rutinose disaccharide at the A ring that lack a methoxy (-OCH3) substitution at the B ring, such as isorhoifolin, clone 1C116 did not secrete IL-2 (Fig. 2C).

**Table 1:** Stimulatory activity of various samples for IL-2 secretion measured from γδ-TCR transfected J.RT3-T3.5 cell-derived clones, 1C116 and 2C21.

Sample	IL-2 secretion from 1C116 (U/mL)	IL-2 secretion from 2C21 (U/mL)
Quercetin	0.81 ± 0.11	0.09 ± 0.04
Isoquercitrin	0.04 ± 0.03	0.03 ± 0.03
Hyperoside	0.05 ± 0.02	0.02 ± 0.02
Galangin	1.15 ± 0.16	0.01 ± 0.01
Kaempferol	0.36 ± 0.02	0.00 ± 0.00
Morin	0.00 ± 0.00	0.00 ± 0.00
Myricetin	0.01 ± 0.00	0.00 ± 0.00

Taken together, these findings clearly show that clone 1C116 is specifically and strongly stimulated through its Vδ1-TCR by flavonoid glycosides having both rutinose at the A ring and methoxy (-OCH3) substitution at the B ring. Furthermore, hesperidin and linarin showed striking stimulatory activity on clone 1C116. Thus, we focused on hesperidin and linarin and examined whether these flavonoid glycosides could suppress the replication of R5-type HIV-1 in CD4^+^ NKT cells through the activation of Vδ1-TCR-expressing Vδ1^+^ T cells.

### Expansion and activation of Vδ1^+^ T cells in the blood stimulated by flavonoid glycosides, hesperidin and linarin

**Figure 3:**
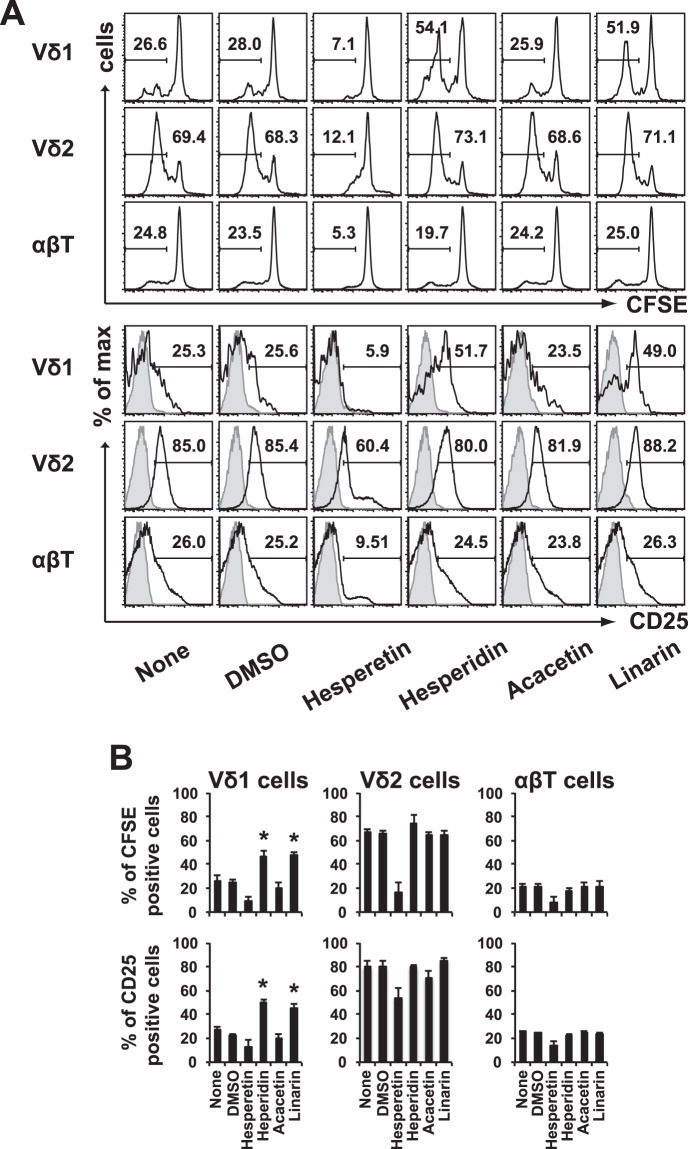
Effects of hesperidin and linarin on human Vδ1^+^ cells in PBMCs. (**A**) CFSE-labeled human PBMCs from a healthy donor were cultured in the presence of 100 µg/mL hesperetin, hesperidin, acacetin, or linarin or 0.01% DMSO for 14 days. CFSE expression (upper) and CD25 expression (lower) in Vδ1^+^ T cells (Vδ1), Vδ2^+^ T cells (Vδ2) and αβ^+^ T cells (αβT) in the PBMC population gated on CD3^+^ are shown. The shaded histogram indicates isotype controls. The experiment was repeated three times, and representative data are shown. (**B**) Statistical analysis was performed. The data are expressed as the mean + SEM of technical replicates in three independent experiments. *P < 0.05, ANOVA with Dunnett’s test.

We first examined whether human Vδ1^+^ T cells can be stimulated to proliferate and undergo functional activation by hesperidin and linarin. To perform this experiment, we first labeled PBMCs with CFSE. Then, the labeled cells were incubated with 100 μg/mL hesperetin, hesperidin, acacetin or linarin, or 0.01% DMSO for 14 days in 48-well plates containing 0.5 mL of CCM with 5% AB human serum and 100 U/mL recombinant IL-2. After culture, the cells were incubated with anti-Vδ1-APC antibody and anti-CD25-PE/Cy7 antibody at 4 °C for 30 min and were analyzed by flow-cytometry. When compared with unstimulated control or vehicle DMSO, hesperetin and acacetin did not significantly stimulate PBMC-derived Vδ1^+^ T cells, but hesperidin and linarin expanded the number of PBMC-derived Vδ1^+^ T cells (Fig. 3A, upper panel and Supplementary Fig. S2). Additionally, hesperidin and linarin activated these cells to express CD25 (Fig. 3A, lower panel and Supplementary Fig. S2). The capacity of flavonoid glycosides to induce proliferation and activation of Vδ1^+^ T cells by was confirmed in repeat experiments (Fig. 3B). In contrast, neither Vδ2^+^ T cells and αβ^+^ T cells responded to hesperidin and linarin (Fig. 3A,B).

### Cytokine and chemokine profiles secreted from Vδ1^+^ T cells stimulated with flavonoid glycosides

**Figure 4:**
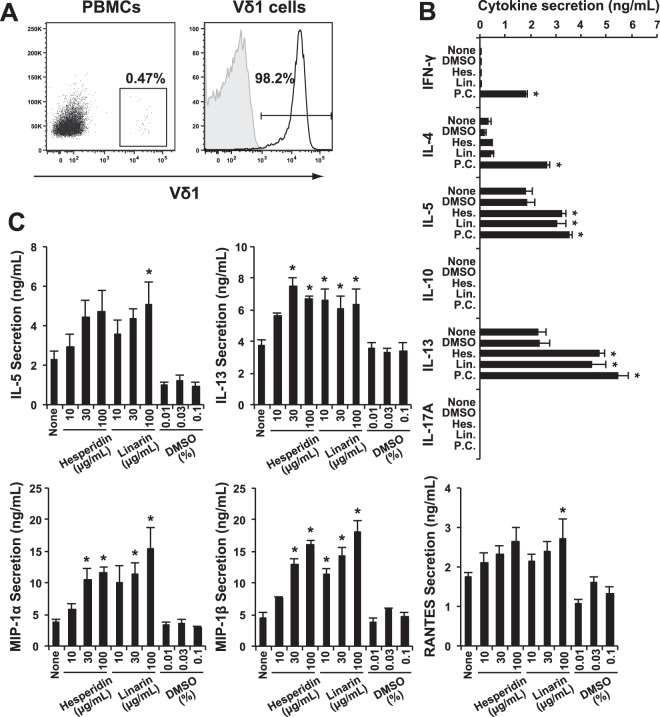
Cytokine and chemokine secretion profiles of resting human Vδ1^+^ T cells obtained from PBMCs stimulated with hesperidin and linarin. (**A**) The percentage of Vδ1^+^ T cells among PBMCs were determined as 0.47%, and the Vδ1^+^ T cells were sorted by FACSAriaII and were expanded with 2 μg/mL PHA and 100 U/mL rIL-2. The open histogram indicates staining with anti-Vδ1 mAb, and the shaded histogram shows the staining with the isotype control. The stained cells were analyzed by FACSCantoII using FlowJo software. (**B**) The cytokine profile of culture supernatant from the resting Vδ1^+^ T cells stimulated with/without 25 ng/mL PMA and 1 μg/mL ionomycin (P.C.), 100 μg/mL hesperidin or linarin, or 0.01% DMSO for 3 days. The amounts of these cytokines were measured by BD™ CBA kit and FACSCantoII. The data are expressed as the mean + SEM (n = 4). *P < 0.05 ANOVA with Dunnett’s test. (**C**) IL-5 and IL-13 (upper) and MIP-1α, MIP-1β and RANTES (lower) amounts of culture supernatant from the resting Vδ1^+^ T cells stimulated with/without hesperidin, linarin, DMSO for 3 days. The amounts of these cytokines and chemokines were measured by using each specific ELISA kit. The data are expressed as the mean + SEM (n = 6). *P < 0.05 determined by using ANOVA with Dunnett’s test.

These experiments revealed that flavonoid glycosides such as hesperidin and linarin can stimulate Vδ1^+^ T cells through their TCRs. We have reported previously that chemokines such as CCL3 (MIP-1α), CCL4 (MIP-1β) and CCL5 (RANTES) secreted by Vδ1^+^ T cells suppress R5-HIV-1-NL(AD8) viral replication in CD4^+^ NKT cells^6^. In addition, they activate Vδ1^+^ T cells through enhanced MICA/MICB expression on NL(AD8)-infected CD4^+^ NKT cells^6^. We, thus, asked whether the flavonoid glycoside-activated Vδ1^+^ T cells would secrete MIP-1α, MIP-1β and RANTES. To perform these experiments, we sorted and expanded Vδ1-TCR expressing T cells from PBMCs (Fig. 4A) and stimulated them with various flavonoid glycosides to examine the cytokine profiles. Surprisingly, we found that the predominant cytokines observed in the supernatant of flavonoid glycoside-activated Vδ1^+^ T cells were IL-5 and IL-13 (Fig. 4B) but not IL-17 and IFN-γ, a profile very close to that of innate lymphocytes 2 (ILC-2) rather than that of γδ T cells^24^. Moreover, we examined the effect of three doses of hesperidin and linarin (10 µg/mL, 30 µg/mL, 100 µg/mL) and measured the amounts of secreted cytokines and chemokines. In comparison with the effect of control DMSO, the secreted amounts of IL-5, IL-13, as well as MIP-1α, MIP-1β and RANTES were all enhanced (Fig. 4C). These findings strongly suggested that PBMCs stimulated by flavonoid glycosides such as hesperidin and linarin seem to suppress R5-HIV-1-NL(AD8) viral replication in CD4^+^ NKT cells.

### Suppression in the replication of R5-type of HIV-1 through Vδ1^+^ T cells activated by flavonoid glycosides, hesperidin and linarin

**Figure 5:**
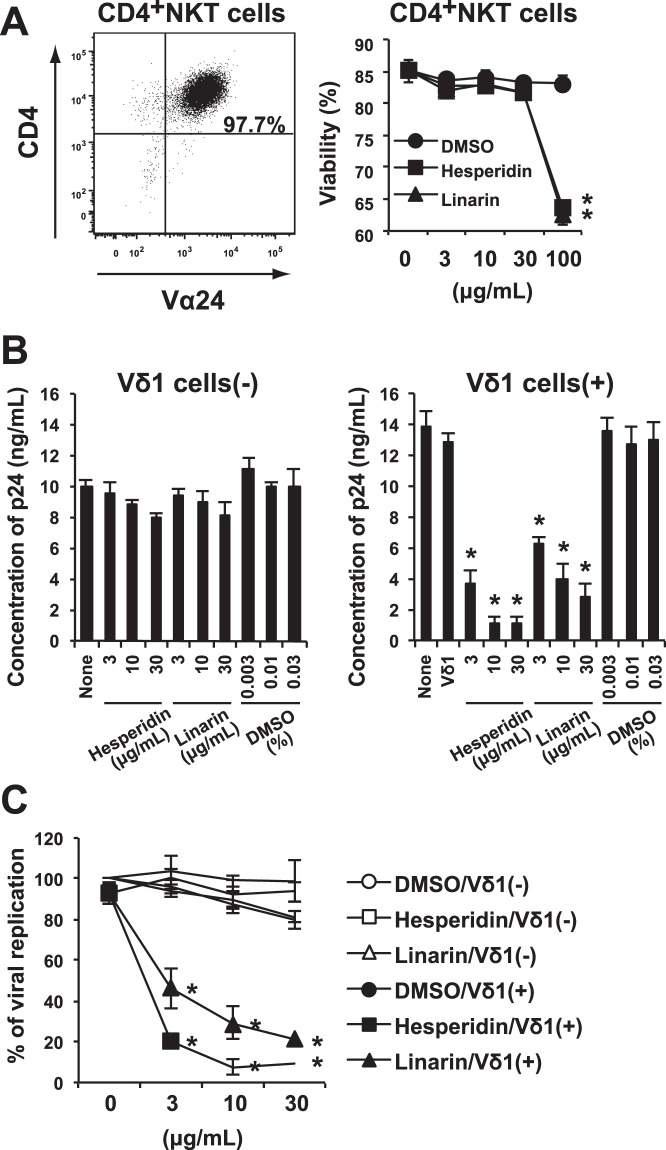
Effects of hesperidin and linarin on the replication of R5-type of HIV-1 in CD4^+^ NKT cells through Vδ1^+^ T cells. (**A**) CD4^+^ NKT cells induced from PBMCs by using α-GalCer (left). Viability of CD4^+^ NKT cells cultured with hesperidin, linarin or DMSO (right). (**B**) As described in Methods, CD4^+^ NKT cells were infected with R5-tropic NL(AD8) HIV-1. Then, HIV-1-infected CD4^+^ NKT cells were cultured in the presence of 3, 10, or 30 μg/mL hesperidin or linarin or 0.003, 0.01 or 0.03% DMSO with (right)/without (left) the resting Vδ1^+^ T cells for 3 days. HIV-1 p24 concentration of the culture supernatant was measured by using specific ELISA kit. (**C**) The percentage of HIV-1 viral replication was also shown. The data are expressed as the mean + SEM of three independent experiments. *P < 0.05 determined by using ANOVA with Dunnett’s test.

CD4^+^ Vα24^+^ NKT cells were induced from human PBMCs (Fig. 5A, left panel) and infected with R5-tropic NL(AD8) HIV-1. The virus-infected NKT cells were cultured with or without Vδ1^+^ T cells stimulated with hesperidin, linarin, or DMSO. Addition of more than 100 μg/mL of hesperidin or linarin to the culture medium for interfered with NKT cell replication/survival, indicating that more than 100 μg/mL was toxic (Fig. 5A, right panel). At lower concentrations that avoided this toxicity, flavonoid glucosides did not suppress R5-HIV-1-NL(AD8) production by infected CD4^+^ NKT cells in the absence of Vδ1^+^ T cells (Fig. 5B, left panel), but a measurable amount of suppression of viral replication was observed in the presence of Vδ1^+^ T cells and the flavonoids (Fig. 5B, right panel and Fig. 5C). Conversely, resting Vδ1^+^ T cells alone did not suppress R5-HIV-1-NL(AD8) production by infected CD4^+^ NKT cells. Taken together, these findings suggest that resting Vδ1^+^ T cells may contribute to control R5 tropic HIV-1 replication in CD4^+^ NKT cells, when stimulated by flavonoid glycosides such as hesperidin and linarin. So far as we know, this is the first demonstration that flavonoid glycoside will activate Vδ1^+^ T cells and yield a functional outcome.

## Discussion

In the present study, we have successfully established two distinct γδ TCR-transfected clones, 1C116 and 2C21, expressing either Vδ1-TCR or Vδ2-TCR, respectively. 2C21 is specifically stimulated to secrete IL-2 through the expressed Vδ2-TCR by any of several previously reported antigenic molecules such as IPP, ethylamine or by amino-bisphosphonates such as risedronate.

Using a similar technical approach, we found that another γδ TCR-transfected clone, 1C116, is specifically stimulated through its Vδ1-TCR by flavonoid glycosides such as hesperidin and linarin, which have both rutinose at the A ring and methoxy (-OCH3) substitution at the B ring. To our knowledge, this is the first demonstration that Vδ1^+^ cells specifically recognize flavonoids, especially flavonoid glycosides. Moreover, the identified flavonoid glycosides not only activated the engineered Jurkat clone expressing Vδ1, but also stimulated PBMC-derived Vδ1^+^ T cells to secrete both IL-5 and IL-13 cytokines as well as the chemokines MIP-1α, MIP-1β and RANTES. Consistent with our prior data, these mediators suppressed R5-HIV-1-NL(AD8) viral replication in CD4^+^ NKT cells. Therefore, the newly identified flavonoid glycosides that stimulate Vδ1^+^ T cells may constitute a new class of anti-HIV drugs able to act in the mucosal compartment to suppress the R5-type of HIV-1.

A number of studies have shown that many plants produce various flavonoids as defense factors against microbes and toxins, offering protection against pathogenic bacteria, fungi and viruses. Although the exact mechanisms behind their anti-microbial properties are not fully understood, two flavonoids, such as hesperidin and its aglycone, hesperetin, showed various biological properties, including anti-oxidant, anti-inflammatory and anti-cancer effects^25^. It should be noted that polyphenols have intensively been studied due to their beneficial effects in both cardiovascular diseases and cancer.

Among polyphenols, quercetin is one of the most studied compounds. It is found in apples, berries, oranges, grapes, onions and tea, and it is metabolized and then absorbed by the intestinal microbiota. High levels of unabsorbed flavonoid compounds in the gut play an important role in the intestine’s health. The remaining flavonoids may suppress the growth of many intestinal pathogenic microbiota. Recently, it was demonstrated that one flavonoid glycoside called quercetin 3-β-O-D-glucoside (Q3G) has the ability to protect mice from Ebola, even when given only 30 min prior to infection^26^. Moreover, a number of reports on flavonoids, including on quercetin and its derivatives, demonstrated anti-viral activities for a variety of viruses, such as influenza virus^27,28^, hepatitis C virus^29^, Chikungunya virus^30^ and Epstein-Barr virus^31^. Therefore, a number of flavonoids having quercetin-like structures will suppress the viral replication of various viruses, but most of these flavonoids will not activate both Vδ1^+^ T cells and Vδ2^+^ T cells. Only a limited repertoire of Vδ1^+^ T cells have a stronger ability to suppress viral replication than the effective flavonoids, and those Vδ1^+^ T cells can be mediated through flavonoid glycosides, such as hesperidin and linarin.

R5-type of HIV-1 replication can be observed in mucosal CD4^+^ NKT cells with the invariant Vα24 TCR, even in individuals given effective HAART-treatment^5^. Our current findings suggest that exposure of the Vδ1^+^ T cells in such patients to flavonoid glycosides such as hesperidin and linarin may contribute to limiting R5-type of HIV-1 replication. Finally, the findings also imply that the treatment of not only a variety of viral infections but also other virus-related diseases such as malignancies or several autoimmune-diseases might be enhanced by activation of host Vδ1^+^ T cells with flavonoid glycosides.

## Methods

### Immortalization of peripheral blood T cells by *Herpesvirus saimiri* (*H*. *saimiri*) and establishment of a γ1δ1^+^ T cell clone

PBMCs were obtained from a healthy individual by gradient centrifugation using Ficoll-Hypaque (GE Healthcare, Uppsala, Sweden). The isolated PBMCs were labeled with anti-pan γδ-TCR mAb (B6.1; BD Bioscience, San Diego, CA), and then γδ T cells were positively selected by FACSAriaII (BD Bioscience, Mountain View, CA). The purified γδ T cells were stimulated in complete culture medium (CCM)^32^ composed of RPMI-1640 (Thermo Fisher Scientific, Waltham, MA) supplemented with 10% heat-inactivated fetal calf serum (FCS) (HyClone, Logan, UT), 5 mM HEPES buffer (Thermo Fisher Scientific), 100 U/ml penicillin (Thermo Fisher Scientific), 100 μg/ml streptomycin (Thermo Fisher Scientific), 2 mM L-glutamine (Thermo Fisher Scientific), 2 mM sodium pyruvate (Thermo Fisher Scientific), 2 mM nonessential amino acids (Thermo Fisher Scientific), 2 mM-modified vitamins (Thermo Fisher Scientific), and 0.5 μM 2-mercaptoethanol (2-ME) (Thermo Fisher Scientific) for 3 days with 1 μg/mL phytohemagglutinin (PHA) (Sigma-Aldrich, St. Louis, MO). Then, the cells were infected with the 488 strain of *H*. *saimiri* subgroup C (a gift of Dr. M. Yasukawa, Ehime University, Japan) as described previously^33^, followed by further culture in the presence of 10 U/mL recombinant interleukin 2 (IL-2) (Shionogi, Osaka, Japan). Single cells were sorted FACSAriaII, resulting in the isolation of γ1δ1^+^ T cell clones showing stable proliferation over several months without mitogenic stimulation. The study was performed in accordance with the Declaration of Helsinki and under the approval of the Review Board of Nippon Medical School, and all human participants gave written informed consent.

### Reconstitution of the γδ-TCR in TCR-deficient cells

Full-length cDNAs encoding the TCR-γ1 and TCR-δ1 were isolated from an immobilized γ1δ1^+^ T cell clone established as described previously^18^. TCR-γ1 cDNA was amplified using the following sense primer, 5′-GGCGGCGGCCGCGAAGGCATGCGGTGGGCCCT-3′, and antisense primer, 5′-GGGCTCGAGCTGTTATGATTTCTCTCCATT-3, and the cDNA was cloned into pEF1/Myc-His A (Thermo Fisher Scientific, Waltham, MA). TCR-δ1 was amplified using sense primer, 5′-GGCGGCGGCCGCCTTCAGGCAGCACAACTC-3′, and antisense primer, 5′-GGGCTCGAGGGAGTGTAGCTTCCTCAT-3′, and it was cloned into pREP4 (Thermo Fisher Scientific). Similarly, full-length cDNAs encoding the TCR-γ2 and TCR-δ2 were isolated from Vδ2-rich T cells, which were induced from PBMCs by repetitive stimulation with 2.5 μg/mL risedronate (Ajinomoto Pharmaceutical. Co., Tokyo, Japan) in the presence of 10 U/mL rIL-2 for 7 days. TCR-γ2 cDNA was amplified using the following sense primer, 5′-GGGCTCGAGGACACCGCTTTACAACGA-3′, and antisense primer 5′-GGGTCTAGAGTGAGGTTCTCTGTGT-3′, and it was cloned into pEF1/Myc-His A. TCR-δ2 was amplified using sense primer, 5′-GGGCTCGAGAACACTTGTGTGTTGGTTCA-3′, and antisense primer, 5′-GGGGGATCCAGTGTATCACTTGTAGGAG-3′, and it was cloned into pREP4.

These constructed plasmids were doubly transfected into the TCR-deficient Jurkat cells, J.RT3-T3.5 (ATCC, Manassas, VA), by electroporation at 240 to 270 V. Two or three days after the transfection, the transfected cells were cultured in the presence of 1 mg/mL G418 sulfate (Thermo Fisher Scientific) and 0.5 mg/mL hygromycin B (FUJIFILM Wako Pure Chemical Corporation, Osaka, Japan) to select γδ TCR-bearing cells. Clones were isolated by limiting the dilution at which drug-resistant cells were plated at 0.5 or 1 cells/well in 96-well microtiter plates, and the cells were analyzed for γδ-TCR expression by flow cytometry. 1C116 and 2C21 clones that stably expressed Vδ1- and Vδ2-TCRs, respectively, were obtained and used in this study.

### Flow cytometric analysis and TCR blocking assays of the γδ-TCR transfected clones

J.RT3-T3.5 and clones 1C116 and 2C21 were incubated with anti-pan-γδ-PE mAb (BioLegend, San Diego, CA), anti-Vδ1-FITC mAb (Miltenyi Biotec GmbH, Bergisch Gladbach, Germany), anti-Vδ2-PE mAb (BioLegend), and anti-CD3-APC mAb (BioLegend). After incubation with the mAbs at 4 °C for 30 min, the treated cells were washed and resuspended in PBS containing 3% FCS and 0.05% NaN_3_ (FACS buffer). Then, the cells were analyzed by FACSCantoII (BD Biosciense) using FlowJo software (BD Biosciense). Additionally, to do the TCR-blocking assay, the established γδ-TCR transfected clones were incubated with 20 μg/mL anti-pan-γδ mAb (clone B1: BioLegend) at room temperature for an hour, and the following stimulation assay was performed.

### Stimulation of the γδ-TCR transfected clones and measurement of the amount of IL-2 by ELISA

In total, 1 × 10^5^ clone 1C116 or 2C21 cells, were cultured with each material (concentration ranges are described below) or vehicle control (dimethyl sulfoxide: DMSO) and 20 ng/mL PMA in 96-well U-bottom tissue culture plates in 200 μL of CCM for 24 h. The materials were 0–100 μg/mL flavonoids (quercetin, isoquercitrin, hyperoside, galangin, kaempferol, morin, myricetin, hesperetin, hesperidin, acacetin, linarin, isorhoifolin, (all purchased from Extrasynthese, Lyon, France)), 0–1000 μM IPP (Sigma-Aldrich), 0–2000 μM risedronate, 0–33.5 μM methylamine (Sigma-Aldrich), 0–33.5 μM ethylamine (Sigma-Aldrich) and 1 μg/mL purified anti-human CD3 mAb (OKT3: Thermo Fisher Scientific). After the incubation, the culture supernatant was corrected, and the concentration of IL-2 was analyzed using an ELISA kit (BD Biosciense).

### Flow cytometric analysis of γδ T cells in PBMCs cultured with flavonoids

PBMCs were labeled with a Cell Trace CFSE Cell Proliferation Kit (carboxy-fluorescein diacetate succinimidyl ester (CFDA-SE, also known as CFSE)) (Thermo Fisher Scientific). Then, the cells were incubated with 100 μg/mL hesperetin, hesperidin, acacetin or linarin or 0.01% DMSO (vehicle control) for 14 days in 48-well plates containing 0.5 mL of CCM containing 5% AB human serum (Biowest, Nuaille, France) and 100 U/mL rIL-2. After they were cultured, the cells were incubated with anti Vδ1-APC mAb, anti Vδ2-PE mAb, anti CD3-APC/Cy7 mAb (BioLegend) and anti CD25-PE/Cy7 mAb (BioLegend) at 4 °C for 30 min. Then, the cells were washed and suspended in FACS buffer and were analyzed by FACSCantoII using FlowJo software.

### Induction of resting Vδ1^+^ T cells from human PBMCs

According to the procedure reported previously^8^, polyclonal Vδ1^+^ T cells freshly obtained from human PBMCs were incubated with 2 μg/mL PHA with 1 × 10^6^/mL irradiated PBMCs and were further cultured for an additional 21 days in 24-well culture plates containing CCM supplemented with 5% AB human serum and 100 U/mL rIL-2, and the medium was half-exchanged every 3–4 days. After the initial culture, the Vδ1^+^ cells were cultured by the same procedure for 14 days to rest.

### Measurement of the amount of cytokine and chemokine production from Vδ1^+^ T cells stimulated by flavonoids

The Vδ1^+^ T cells (5 × 10^4^) were cultured in 200 μl of CCM supplemented with 5% AB human serum and 100 U/mL rIL-2 in round-bottom 96-well plates. In the cell cultures, 25 ng/mL PMA and 1 μg/mL ionomycin (Sigma-Aldrich), 10, 30 or 100 μg/mL hesperidin or linarin or 0.01, 003 or 0.1% DMSO were added into the well. After 3 days, 150 μl of culture supernatant was collected from each well and stored at −80 °C until the measurement of cytokines and chemokines.

For the quantification of multiple cytokines (IFN-γ, IL-4, IL-5, IL-10, IL-13 and IL-17A), BD™ CBA kit (BD Bioscience) and FACSCantoII were used. Moreover, the amounts of cytokines such as IL-5 and IL-13 were determined by using an IL-5 ELISA kit (R&D Systems, Minneapolis, MN) and an IL-13 ELISA kit (Thermo Fisher Scientific), respectively. Additionally, chemokines such as MIP-1α, MIP-1β and RANTES in the medium were quantified by using each ELISA kit (R&D Systems).

### Induction of CD4^+^ NKT cells from human PBMCs and assessment of cell viability when cultured with hesperidin or linarin

NKT cells^4^ were induced from PBMCs by using α-galactosylceramide (α-GalCer; KRN7000: Funakoshi Co., Ltd., Tokyo, Japan) and sorted into CD4^+^ Vα24^+^ population^6^ by using FACSAriaII. The cells were stimulated with 2 μg/mL of PHA in CCM containing 20 U/mL of IL-2 for 3 days. For the assessment of cell viability, the NKT cells (1 × 10^4^) were cultured with 3, 10, 30 or 100 μg/mL hesperidin, linarin or 0.003, 0.01, 003 or 0.1% DMSO for 3 days. After the culture, the cell viability was calculated by the trypan blue exclusion test.

### Infection of CD4^+^ NKT cells with NL(AD8) HIV-1 (R5-type) and analysis of the effect of flavonoids on virus replication

The CD4^+^ NKT cells (1–2 × 10^5^) were infected with NL(AD8) HIV-1 (R5-type)^34^ at 0.1 MOI for 2 h at 37 °C in the presence of 30 μg/mL DEAE-Dextran (Sigma-Aldrich). After the NL(AD8)-infected cells were washed extensively three times with RPMI-1640 containing 2% FCS, they were further incubated in CCM containing 20 U/ml of rIL-2 in round-bottom 96-well plate.

Then, 1 × 10^4^ NL(AD8)-infected NKT cells were cultured with or without resting 1 × 10^4^ Vδ1^+^ cells in 200 μl of CCM containing 20 U/mL IL-2 in round-bottom 96-well plates. In the cell cultures, 3, 10 or 30 μg/mL hesperidin or linarin or 0.003, 001 or 0.03% DMSO (vehicle control) was added into the well. On day 3, after the cultures were initiated, 100 μl of culture supernatant was collected from each well and stored at −80 °C until the measurement of p24 antigen. HIV-1 capsid protein p24 production in the culture supernatant was also measured using the ELISA kit (Sino Biological, Beijing. China).

### Statistical analyses

The results were analyzed using an ANOVA with Dunnett’s post hoc test, and were presented as the mean ± SEM. Differences at *p* < 0.05 were considered significant. The statistical analysis was performed using Statview 5.0 software (SAS Institute Inc., Cary, NC).

### Study approval

The study was conducted in accordance with the guidelines of the Declaration of Helsinki and principles of Good Clinical Practice and approved by the Review Board of Nippon Medical School, and all human participants gave written informed consent.

## Supplementary information

Supplementary Figures

## References

[1] Squires K (2016). Integrase inhibitor versus protease inhibitor based regimen for HIV-1 infected women (WAVES): a randomised, controlled, double-blind, phase 3 study. Lancet HIV.

[2] Finzi D (1997). Identification of a reservoir for HIV-1 in patients on highly active antiretroviral therapy. Science.

[3] Chun TW (2000). Relationship between pre-existing viral reservoirs and the re-emergence of plasma viremia after discontinuation of highly active anti-retroviral therapy. Nat Med.

[4] Kawano T (1997). CD1d-restricted and TCR-mediated activation of valpha14 NKT cells by glycosylceramides. Science.

[5] Matsumura J (2014). A possible origin of emerged HIV-1 after interrupting anti-retroviral therapy. Biomed Res.

[6] Omi K (2014). Inhibition of R5-tropic HIV type-1 replication in CD4(+) natural killer T cells by gammadelta T lymphocytes. Immunology.

[7] Motsinger A (2002). CD1d-restricted human natural killer T cells are highly susceptible to human immunodeficiency virus 1 infection. J Exp Med.

[8] Shamshiev, A. *et al*. Self glycolipids as T-cell autoantigens. *Eur J Immunol* **29**, 1667–1675, doi:10.1002/(SICI)1521-4141(199905)29:05<1667::AID-IMMU1667>3.0.CO;2-U (1999). 10.1002/(SICI)1521-4141(199905)29:05<1667::AID-IMMU1667>3.0.CO;2-U 10359121

[9] Tanaka Y (1995). Natural and synthetic non-peptide antigens recognized by human gamma delta T cells. Nature.

[10] Das H, Wang L, Kamath A, Bukowski JF (2001). Vgamma2Vdelta2 T-cell receptor-mediated recognition of aminobisphosphonates. Blood.

[11] Bukowski JF, Morita CT, Brenner MB (1999). Human gamma delta T cells recognize alkylamines derived from microbes, edible plants, and tea: implications for innate immunity. Immunity.

[12] Uldrich AP (2013). CD1d-lipid antigen recognition by the gammadelta TCR. Nat Immunol.

[13] Roy S (2016). Molecular Analysis of Lipid-Reactive Vdelta1 gammadelta T Cells Identified by CD1c Tetramers. J Immunol.

[14] Xu B (2011). Crystal structure of a gammadelta T-cell receptor specific for the human MHC class I homolog MICA. Proc Natl Acad Sci USA.

[15] Hayday AC (2000). [gamma][delta] cells: a right time and a right place for a conserved third way of protection. Annu Rev Immunol.

[16] Boismenu R, Havran WL (1997). An innate view of gamma delta T cells. Curr Opin Immunol.

[17] Chien YH, Konigshofer Y (2007). Antigen recognition by gammadelta T cells. Immunol Rev.

[18] Narazaki, H. *et al*. Perforin-dependent killing of tumor cells by Vgamma1Vdelta1-bearing T-cells. *Immunol Let*t **8**6, 113–119, S0165247802002924 [pii] (2003). 10.1016/s0165-2478(02)00292-4 12600753

[19] Bukowski JF (1995). V gamma 2V delta 2 TCR-dependent recognition of non-peptide antigens and Daudi cells analyzed by TCR gene transfer. J Immunol.

[20] Vavassori S (2013). Butyrophilin 3A1 binds phosphorylated antigens and stimulates human gammadelta T cells. Nat Immunol.

[21] Kamath AB (2003). Antigens in tea-beverage prime human Vgamma 2Vdelta 2 T cells *in vitro* and *in vivo* for memory and nonmemory antibacterial cytokine responses. Proc Natl Acad Sci USA.

[22] Liu RH (2004). Potential synergy of phytochemicals in cancer prevention: mechanism of action. J Nutr.

[23] Panche AN, Diwan AD, Chandra SR (2016). Flavonoids: an overview. J Nutr Sci.

[24] Kohlgruber AC (2018). Gammadelta T cells producing interleukin-17A regulate adipose regulatory T cell homeostasis and thermogenesis. Nat Immunol.

[25] Iranshahi M, Rezaee R, Parhiz H, Roohbakhsh A, Soltani F (2015). Protective effects of flavonoids against microbes and toxins: The cases of hesperidin and hesperetin. Life Sci.

[26] Qiu X (2016). Prophylactic Efficacy of Quercetin 3-beta-O-d-Glucoside against Ebola Virus Infection. Antimicrob Agents Chemother.

[27] Kim Y, Narayanan S, Chang KO (2010). Inhibition of influenza virus replication by plant-derived isoquercetin. Antiviral Res.

[28] Abdal Dayem A, Choi HY, Kim YB, Cho SG (2015). Antiviral effect of methylated flavonol isorhamnetin against influenza. PLoS One.

[29] Khachatoorian R (2012). Divergent antiviral effects of bioflavonoids on the hepatitis C virus life cycle. Virology.

[30] Lani R (2015). Antiviral activity of silymarin against chikungunya virus. Sci Rep.

[31] Lee M (2015). Quercetin-induced apoptosis prevents EBV infection. Oncotarget.

[32] Takahashi H (1996). Inactivation of human immunodeficiency virus (HIV)-1 envelope-specific CD8^+^ cytotoxic T lymphocytes by free antigenic peptide: a self-veto mechanism?. J Exp Med.

[33] Yasukawa M, Inoue Y, Kimura N, Fujita S (1995). Immortalization of human T cells expressing T-cell receptor gamma delta by herpesvirus saimiri. J Virol.

[34] Englund G, Theodore TS, Freed EO, Engelman A, Martin MA (1995). Integration is required for productive infection of monocyte-derived macrophages by human immunodeficiency virus type 1. J Virol.

