# Supplementary material for: Suppression of R5-type of HIV-1 in CD4+ NKT cells by Vδ1+ T cells activated by flavonoid glycosides, hesperidin and linarin
Source: Sci Rep. 2019 May 17;9:7506. doi: 10.1038/s41598-019-40587-6 (PMC6525194; doi:10.1038/s41598-019-40587-6)
Supplement: Supplementary file 1 — Supplementary Figures [file 41598_2019_40587_MOESM1_ESM.pdf]

Suppression of R5-type of HIV-1 in CD4$^+$ NKT cells by Vδ1$^+$ T cells activated by flavonoid glycosides, hesperidin and linarin

Michiyuki Yonekawa, Masumi Shimizu, Atsushi Kaneko, Jiro Matsumura, and Hidemi Takahashi

Supplementary Figures

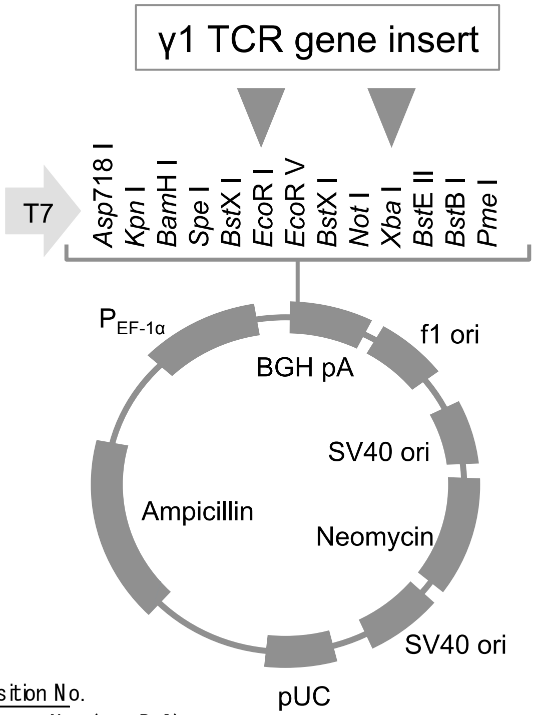

pEF1/Myc-His A plasmid

The sequence for pEF1/Myc-His A (Thermo Fisher Scientific, Cat. No.: V921-20) is available for downloading from www.invitrogen.com.

Composition:
L - Vγ1.4 - N - Jγ2.3 - Cγ2

Boxed sequence: Restriction enzyme site
Gray area: Vγ1.4 region (TCRGV1S4)
Dash line: -N- region
Solid line: Jγ2.3 region (TCRGJ2S2)

```
Position No.
P.    N.  (EcoR I)
       1  GAATTCCGAAGGC    ATG CGG TGG GCC CTA GCG GTG CTT CTA GCT TTC CTG TCT CCT GCC AGT
 1                         M   R   W   A   L   A   V   L   L   A   F   L   S   P   A   S
      62  CAG AAA TCT TCC AAC TTG GAA GGG AGA ACG AAG TCA GTC ATC AGG CAG ACT GGG TCA TCT
17         Q   K   S   S   N   L   E   G   R   T   K   S   V   I   R   Q   T   G   S   S
     122  GCT GAA ATC ACT TGT GAT CTT GCT GAA GGA AGT ACC GGC TAC ATC CAC TGG TAC CTA CAC
37         A   E   I   T   C   D   L   A   E   G   S   T   G   Y   I   H   W   Y   L   H
     182  CAG GAG GGG AAG GCC CCA CAG CGT CTT CTG TAC TAT GAC TCC TAC ACC TCC AGC GTT GTG
57         Q   E   G   K   A   P   Q   R   L   L   Y   Y   D   S   Y   T   S   S   V   V
     242  TTG GAA TCA GGA ATC AGC CCA GGG AAG TAT GAT ACT TAT GGA AGC ACA AGG AAG AAC TTG
77         L   E   S   G   I   S   P   G   K   Y   D   T   Y   G   S   T   R   K   N   L
     302  AGA ATG ATA CTG CGA AAT CTT ATT GAA AAT GAC TCT GGA GTC TAT TAC TGT GCC ACC TGG
97         R   M   I   L   R   N   L   I   E   N   D   S   G   V   Y   Y   C   A   T   W
     362  GAT GGA GAT CTT TAT TAT AAG AAA CTC TTT GGC AGT GGA ACA ACA CTT GTT GTC ACA GAT
117        D   G   D   L   Y   Y   K   K   L   F   G   S   G   T   T   L   V   V   T   D
     422  AAA CAA CTT GAT GCA GAT GTT TCC CCC AAG CCC ACT ATT TTT CTT CCT TCG ATT GCT GAA
137        K   Q   L   D   A   D   V   S   P   K   P   T   I   F   L   P   S   I   A   E
     482  ACA AAA CTC CAG AAG GCT GGA ACA TAC CTT TGT CTT CTT GAG AAA TTT TTC CCA GAT ATT
157        T   K   L   Q   K   A   G   T   Y   L   C   L   L   E   K   F   F   P   D   I
     542  ATT AAG ATA CAT TGG CAA GAA AAG AAG AGC AAC ACG ATT CTG GGA TCC CAG GAG GGG AAC
177        I   K   I   H   W   Q   E   K   K   S   N   T   I   L   G   S   Q   E   G   N
     602  ACC ATG AAG ACT AAC GAC ACA TAC ATG AAA TTT AGC TGG TTA ACG GTG CCA GAA GAG TCA
197        T   M   K   T   N   D   T   Y   M   K   F   S   W   L   T   V   P   E   E   S
     662  CTG GAC AAA GAA CAC AGA TGT ATC GTC AGA CAT GAG AAT AAT AAA AAC GGA ATT GAT CAA
217        L   D   K   E   H   R   C   I   V   R   H   E   N   N   K   N   G   I   D   Q
     722  GAA ATT ATC TTT CCT CCA ATA AAG ACA GAT GTC ACC ACA GTG GAT CCC AAA TAC AAT TAT
237        E   I   I   F   P   P   I   K   T   D   V   T   T   V   D   P   K   Y   N   Y
     782  TCA AAG GAT GCA AAT GAT GTC ATC ACA ATG GAT CCC AAA GAC AAT TGG TCA AAA GAT GCA
257        S   K   D   A   N   D   V   I   T   M   D   P   K   D   N   W   S   K   D   A
     842  AAT GAT ACA CTA CTG CTG CAG CTC ACA AAC ACC TCT GCA TAT TAC ATG TAC CTC CTC CTG
277        N   D   T   L   L   L   Q   L   T   N   T   S   A   Y   Y   M   Y   L   L   L
     902  CTC CTC AAG AGT GTG GTC TAT TTT GCC ATC ATC ACC TGC TGT CTG CTT GGA AGA ACG GCT
297        L   L   K   S   V   V   Y   F   A   I   I   T   C   C   L   L   G   R   T   A
     962  TTC TGC TGC AAT GGA GAG AAA TCA TAA CAGTCTAGA
317        F   C   C   N   G   E   K   S   ·      (Xba I)
```

Supplementary Figure S1A.
Nucleotide and deduced amino acid sequences of γ1 TCR gene in γ1/pEF1 plasmid.

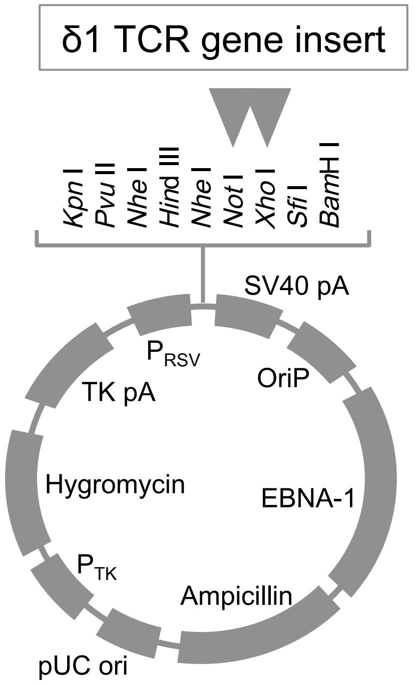

pREP4 plasmid

The sequence for pREP4 (Thermo Fisher Scientific, Cat. No.: V004-50) is available for downloading from www.invitrogen.com.

Composition:
L - Vδ1 - NDN - Jδ1 - Cδ1

Boxed sequence: Restriction enzyme site
Gray area: Vδ1 region (TCRDV101S1)
Dash line: -NDN- region
Solid line: Jδ1 region (TCRDJ1S1)

Position No.

```
P.  N.  (Not I)
  1  GCGGCCGCCTTCAGGCAGCACAACTCACATTTGTGCAAAGAGCTACATGCCAC ATG CTG TTC TCC AGC CTG
1                                                           M   L   F   S   S   L
 72  CTG TGT GTA TTT GTG GCC TTC AGC TAC TCT GGA TCA AGT GTG GCC CAG AAG GTT ACT CAA
7     L   C   V   F   V   A   F   S   Y   S   G   S   S   V   A   Q   K   V   T   Q
132  GCC CAG TCA TCA GTA TCC ATG CCA GTG AGG AAA GCA GTC ACC CTG AAC TGC CTG TAT GAA
27    A   Q   S   S   V   S   M   P   V   R   K   A   V   T   L   N   C   L   Y   E
192  ACA AGT TGG TGG GCA TAT TAT ATT TTT TGG TAC AAG CAA CTT CCC AGC AAA GAG ATG ATT
47    T   S   W   W   A   Y   Y   I   F   W   Y   K   Q   L   P   S   K   E   M   I
252  TTC CTT ATT CGC CAG GGT TCT GAT GAA CAG AAT GCA AAA AGT GGT CGC TAT TCT GTC AAC
67    F   L   I   R   Q   G   S   D   E   Q   N   A   K   S   G   R   Y   S   V   N
312  TTC AAG AAA GCA GCG AAA TCC GTC GCC TTA ACC ATT TCA GCC TTA CAG CTA GAA GAT TCA
87    F   K   K   A   A   K   S   V   A   L   T   I   S   A   L   Q   L   E   D   S
372  GCA AAG TAC TTT TGT GCT CTT GGG GAA CCC GGA GGG TTC CCT CGT ACT GGG GGG ATA GGG
107   A   K   Y   F   C   A   L   G   E   P   G   G   F   P   R   T   G   G   I   G
432  TGG ACC GAT AAA CTC ATC TTT GGA AAA GGA ACC CGT GTG ACT GTG GAA CCA AGA AGT CAG
127   W   T   D   K   L   I   F   G   K   G   T   R   V   T   V   E   P   R   S   Q
492  CCT CAT ACC AAA CCA TCC GTT TTT GTC ATG AAA AAT GGA ACA AAT GTC GCT TGT CTG GTG
147   P   H   T   K   P   S   V   F   V   M   K   N   G   T   N   V   A   C   L   V
552  AAG GAA TTC TAC CCC AAG GAT ATA AGA ATA AAT CTC GTG TCA TCC AAG AAG ATA ACA GAG
167   K   E   F   Y   P   K   D   I   R   I   N   L   V   S   S   K   K   I   T   E
612  TTT GAT CCT GCT ATT GTC ATC TCT CCC AGT GGG AAG TAC AAT GCT GTC AAG CTT GGT AAA
187   F   D   P   A   I   V   I   S   P   S   G   K   Y   N   A   V   K   L   G   K
672  TAT GAA GAT TCA AAT TCA GTG ACA TGT TCA GTT CAA CAC GAC AAT AAA ACT GTG CAC TCC
207   Y   E   D   S   N   S   V   T   C   S   V   Q   H   D   N   K   T   V   H   S
732  ACT GAC TTT GAA GTG AAG ACA GAT TCT ACA GAT CAC GTA AAA CCA AAG GAA ACT GAA AAC
227   T   D   F   E   V   K   T   D   S   T   D   H   V   K   P   K   E   T   E   N
792  ACA AAG CAA CCT TCA AAG AGC TGC CAT AAA CCC AAA GCC ATA GTT CAT ACC GAG AAG GTG
247   T   K   Q   P   S   K   S   C   H   K   P   K   A   I   V   H   T   E   K   V
852  AAC ATG ATG TCC CTC ACA GTG CTT GGG CTA CGA ATG CTG TTT GCA AAG ACT GTT GCC GTC
267   N   M   M   S   L   T   V   L   G   L   R   M   L   F   A   K   T   V   A   V
912  AAT TTT CTC TTG ACT GCC AAG TTA TTT TTC TTG TAA GGCTGACTGGCATGAGGAAGCTACACTC
287   N   F   L   L   T   A   K   L   F   F   L   ·
979  CCTCGAG
     (Xho I)
```

Supplementary Figure S1B.
Nucleotide and deduced amino acid sequences of δ1 TCR gene in δ1/pREP4 plasmid.

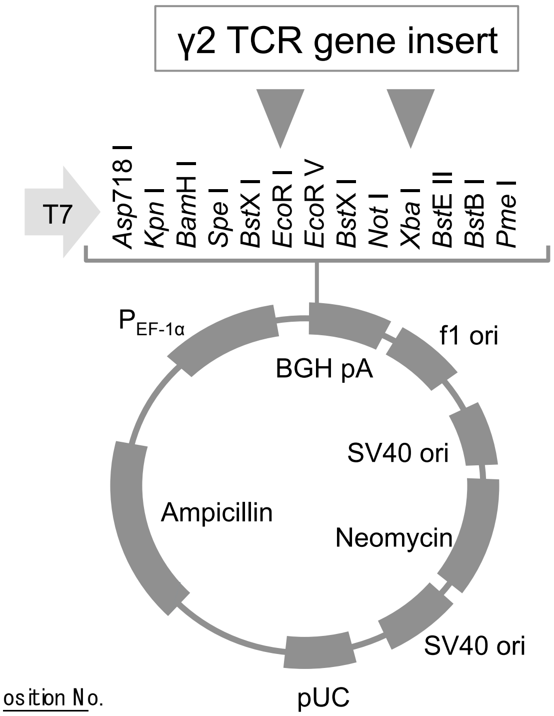

pEF1/Myc-His A plasmid

The sequence for pEF1/Myc-His A (Thermo Fisher Scientific, Cat. No.: V921-20) is available for downloading from www.invitrogen.com.

Composition:
L - Vγ2 - N - Jγ1.2 - Cγ1

Boxed sequence: Restriction enzyme site
Gray area: Vγ2 region (TCRGV2S1)
Dash line: -N- region
Solid line: Jγ1.2 region (TCRGJ1S2)

Position No.

```
P.   N.  (EcoR I)
     1  GAATTCGCC   ATG CTG TCA CTG CTC CAC GCA TCA ACG CTG GCA GTC CTT GGG GCT CTG TGT
1                  M   L   S   L   L   H   A   S   T   L   A   V   L   G   A   L   C
    61  GTA TAT GGT GCA GGT CAC CTA GAG CAA CCT CAA ATT TCC AGT ACT AAA ACG CTG TCA AAA
18       V   Y   G   A   G   H   L   E   Q   P   Q   I   S   S   T   K   T   L   S   K
   121  ACA GCC CGC CTG GAA TGT GTG GTG TCT GGA ATA ACA ATT TCT GCA ACA TCT GTA TAT TGG
38       T   A   R   L   E   C   V   V   S   G   I   T   I   S   A   T   S   V   Y   W
   181  TAT CGA GAG AGA CCT GGT GAA GTC ATA CAG TTC CTG GTG TCC ATT TCA TAT GAC GGC ACT
58       Y   R   E   R   P   G   E   V   I   Q   F   L   V   S   I   S   Y   D   G   T
   241  GTC AGA AAG GAA TCC GGC ATT CCG TCA GGC AAA TTT GAG GTG GAT AGG ATA CCT GAA ACG
78       V   R   K   E   S   G   I   P   S   G   K   F   E   V   D   R   I   P   E   T
   301  TCT ACA TCC ACT CTC ACC ATT CAC AAT GTA GAG AAA CAG GAC ATA GCT ACC TAC TAC TGT
98       S   T   S   T   L   T   I   H   N   V   E   K   Q   D   I   A   T   Y   Y   C
   361  GCC TTG TGG GAG GAT CAA GAG TTG GGC AAA AAA ATC AAG GTA TTT GGT CCC GGA ACA AAG
118      A   L   W   E   D   Q   E   L   G   K   K   I   K   V   F   G   P   G   T   K
   421  CTT ATC ATT ACA GAT AAA CAA CTT GAT GCA GAT GTT TCC CCC AAG CCC ACT ATT TTT CTT
138      L   I   I   T   D   K   Q   L   D   A   D   V   S   P   K   P   T   I   F   L
   481  CCT TCA ATT GCT GAA ACA AAG CTC CAG AAG GCT GGA ACA TAC CTT TGT CTT CTT GAG AAA
158      P   S   I   A   E   T   K   L   Q   K   A   G   T   Y   L   C   L   L   E   K
   541  TTT TTC CCT GAT GTT ATT AAG ATA CAT TGG GAA GAA AAG AAG AGC AAC ACG ATT CTG GGA
178      F   F   P   D   V   I   K   I   H   W   E   E   K   K   S   N   T   I   L   G
   601  TCC CAG GAG GGG AAC ACC ATG AAG ACT AAT GAC ACA TAC ATG AAA TTT AGC TGG TTA ACG
198      S   Q   E   G   N   T   M   K   T   N   D   T   Y   M   K   F   S   W   L   T
   661  GTG CCA GAA AAG TCA CTG GAC AAA GAA CAC AGA TGT ATC GTC AGA CAT GAG AAT AAT AAA
218      V   P   E   K   S   L   D   K   E   H   R   C   I   V   R   H   E   N   N   K
   721  AAC GGA GTT GAT CAA GAA ATT ATC TTT CCT CCA ATA AAG ACA GAT GTC ATC ACA ATG GAT
238      N   G   V   D   Q   E   I   I   F   P   P   I   K   T   D   V   I   T   M   D
   781  CCC AAA GAC AAT TGT TCA AAA GAT GCA AAT GAT ACA CTA CTG CTG CAG CTC ACA AAC ACC
258      P   K   D   N   C   S   K   D   A   N   D   T   L   L   L   Q   L   T   N   T
   841  TCT GCA TAT TAC ACG TAC CTC CTC CTG CTC CTC AAG AGT GTG GTC TAT TTT GCC ATC ATC
278      S   A   Y   Y   T   Y   L   L   L   L   L   K   S   V   V   Y   F   A   I   I
   901  ACC TGC TGT CTG CTT AGA AGA ACG GCT TTC TGC TGC AAT GGA GAG AAA TCA TAA TCTAGA
298      T   C   C   L   R   R   T   A   F   C   C   N   G   E   K   S   ·    (Xba I)
```

Supplementary Figure S1C.
Nucleotide and deduced amino acid sequences of γ2 TCR gene in γ2/pEF1 plasmid.

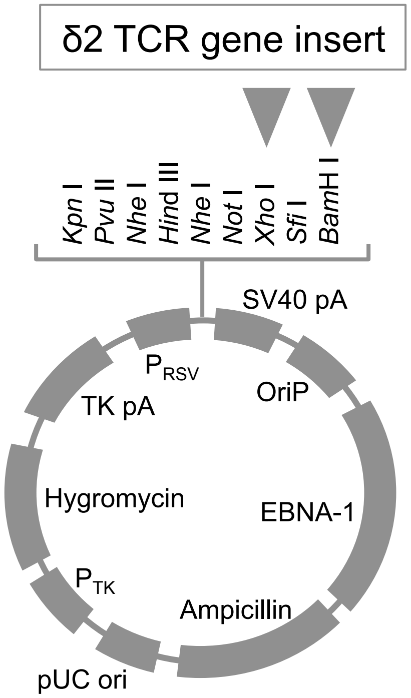

pREP4 plasmid

The sequence for pREP4 (Thermo Fisher Scientific, Cat. No.: V004-50) is available for downloading from www.invitrogen.com.

Composition:
L - Vδ2 - NDN - Jδ1 - Cδ1

Boxed sequence: Restriction enzyme site
Gray area: Vδ2 region (TCRDV102S1)
Dash line: -NDN- region
Solid line: Jδ1 region (TCRDJ1S1)

```
Position No.
P.    N.  (Xho I)
       1  CTCGAGAACACTTGTGTGTTGGTTCAGAGGAGGGACCAGGCAGAAGGTGGTTGAGAGGCAGAGCTGCCCCTGAGT
      76  GAGCC    ATG CAG AGG ATC TCC TCC CTC ATC CAT CTC TCT CTC TTC TGG GCA GGA GTC ATG
1                   M   Q   R   I   S   S   L   I   H   L   S   L   F   W   A   G   V   M
     135  TCA GCC ATT GAG TTG GTG CCT GAA CAC CAA ACA GTG CCT GTG TCA ATA GGG GTC CCT GCC
19         S   A   I   E   L   V   P   E   H   Q   T   V   P   V   S   I   G   V   P   A
     195  ACC CTC AGG TGC TCC ATG AAA GGA GAA GCG ATC GGT AAC TAC TAT ATC AAC TGG TAC AGG
39         T   L   R   C   S   M   K   G   E   A   I   G   N   Y   Y   I   N   W   Y   R
     255  AAG ACC CAA GGT AAC ACA ATC ACT TTC ATA TAC CGA GAA AAG GAC ATC TAT GGC CCT GGT
59         K   T   Q   G   N   T   I   T   F   I   Y   R   E   K   D   I   Y   G   P   G
     315  TTC AAA GAC AAT TTC CAA GGT GAC ATT GAT ATT GCA AAG AAC CTG GCT GTA CTT AAG ATA
79         F   K   D   N   F   Q   G   D   I   D   I   A   K   N   L   A   V   L   K   I
     375  CTT GCA CCA TCA GAG AGA GAT GAA GGG TCT TAC TAC TGT GCC TGT GAC ACC CTG GTT GCT
99         L   A   P   S   E   R   D   E   G   S   Y   Y   C   A   C   D   T   L   V   A
     435  GGG GGT TTG TAC ACC GAT AAA CTC ATC TTT GGA AAA GGA ACC CGT GTG ACT GTG GAA CCA
119        G   G   L   Y   T   D   K   L   I   F   G   K   G   T   R   V   T   V   E   P
     495  AGA AGT CAG CCT CAT ACC AAA CCA TCC GTT TTT GTC ATG AAA AAT GGA ACA AAT GTC GCT
139        R   S   Q   P   H   T   K   P   S   V   F   V   M   K   N   G   T   N   V   A
     555  TGT CTG GTG AAG GAA TTC TAC CCC AAG GAT ATA AGA ATA AAT CTC GTG TCA TCC AAG AAG
159        C   L   V   K   E   F   Y   P   K   D   I   R   I   N   L   V   S   S   K   K
     615  ATA ACA GAG TTT GAT CCT GCT ATT GTC ATC TCT CCC AGT GGG AAG TAC AAT GCT GTC AAG
179        I   T   E   F   D   P   A   I   V   I   S   P   S   G   K   Y   N   A   V   K
     675  CTT GGT AAA TAT GAA GAT TCA AAT TCA GTG ACA TGT TCA GTT CAA CAC GAC AAT AAA ACT
199        L   G   K   Y   E   D   S   N   S   V   T   C   S   V   Q   H   D   N   K   T
     735  GTG CAC TCC ACT GAC TTT GAA GTG AAG ACA GAT TCT ACA GAT CAC GTA AAA CCA AAG GAA
219        V   H   S   T   D   F   E   V   K   T   D   S   T   D   H   V   K   P   K   E
     795  ACT GAA AAC ACA AAG CAA CCT TCA AAG AGC TGC CAT AAA CCC AAA GCC ATA GTT CAT ACC
239        T   E   N   T   K   Q   P   S   K   S   C   H   K   P   K   A   I   V   H   T
     855  GAG AAG GTG AAC ATG ATG TCC CTC ACA GTG CTT GGG CTA CGA ATG CTG TTT GCA AAG ACT
259        E   K   V   N   M   M   S   L   T   V   L   G   L   R   M   L   F   A   K   T
     915  GTT GCC GTC AAT TTT CTC TTG ACT GCC AAG TTA TTT TTC TTG TAA GGCTGACTGGCATGAGGA
279        V   A   V   N   F   L   L   T   A   K   L   F   F   L   ·
     978  AGCTACACTCCTGAAGAAACCAAAGGCTTACAAAAATGCATCTCCTTGGCTTCTGACTTCTTTGTGATTCAAGTTG
    1054  ACCTGTCATAGCCTTGTTAAAATGGCTGCTAGCCAAACCACTTTTTCTTCAAAGACAACAAACCCAGCTCATCCTC
    1130  CAGCTTGATGGGAAGACAAAAGTCCTGGGGAAGGGGGGTTTATGTCCTAACTGCTTTGTATGCTGTTTTATAAAGG
    1206  GATAGAAGGATATAAAAAGATATAGGACTCTTTTTTTACTCCTACAAGTGATACCCTGGATCC
                                                                  (BamH I)
```

Supplementary Figure S1D.
Nucleotide and deduced amino acid sequences of δ2 TCR gene in δ2/pREP4 plasmid.

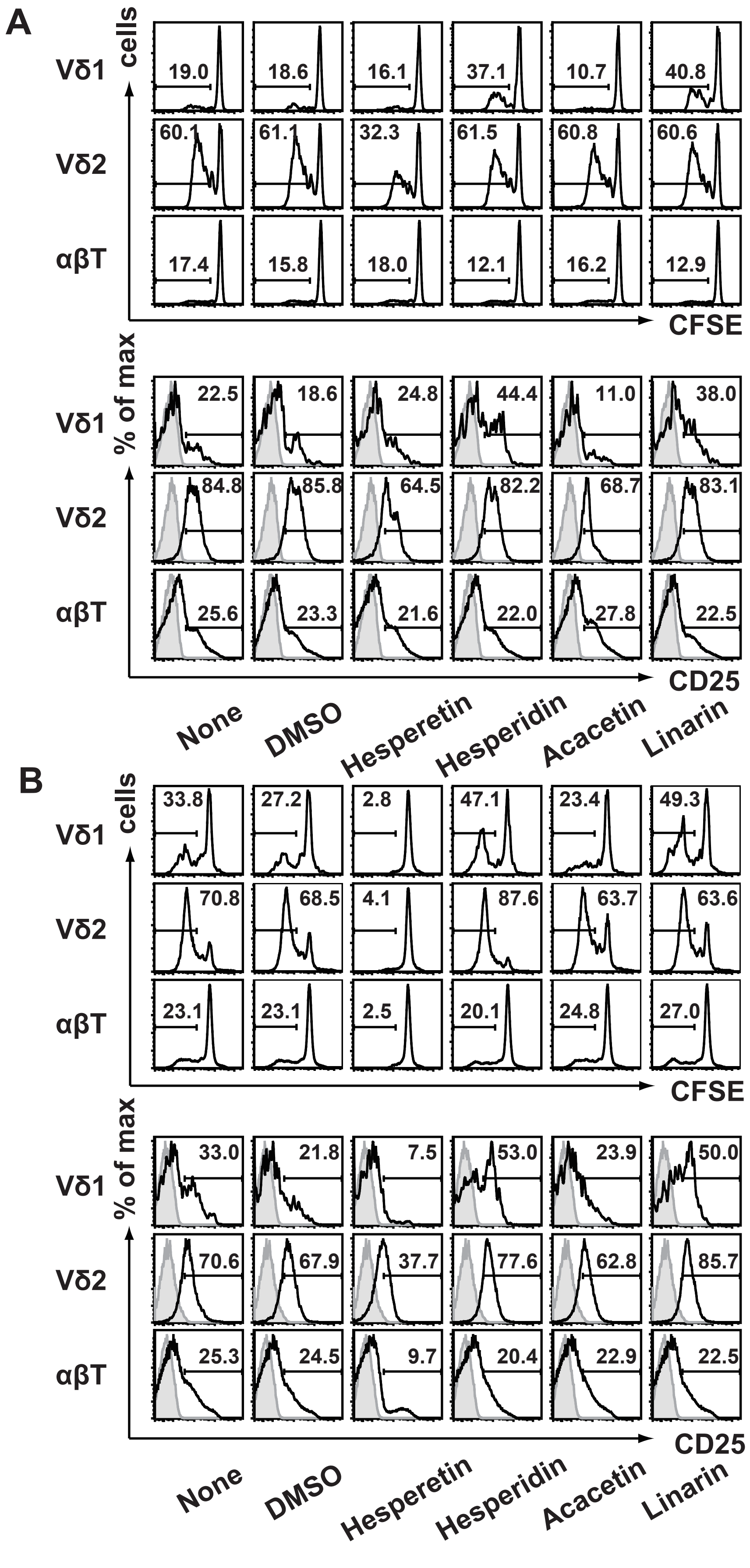

Supplementary Figure S2. Effects of hesperidin and ilinarin on the proliferation and activation of human PBMC-derived Vδ1+ T cells. The data shown as (A) and (B) are technical replicates of a single donor.
